# Association of miRNA-146a rs2910164 and miRNA-196 rs11614913 polymorphisms in patients with ulcerative colitis: A meta-analysis and review

**DOI:** 10.1097/MD.0000000000012294

**Published:** 2018-09-28

**Authors:** Zhongyi Li, Yao Wang, Yi Zhu

**Affiliations:** a Department of Obstetrics and Gynecology, The First Affiliated Hospital of Jinan University; b Department of Epidemiology, Medical School of Jinan University, Guangzhou, Guangdong Province; c Department of Gastroenterological Surgery, First Hospital of Jiaxing, Jiaxing, Zhejiang, China.

**Keywords:** meta-analysis, polymorphisms, ulcerative colitis

## Abstract

**Background:**

It has been reported that the single nucleotide polymorphisms (SNPs) miRNA-196 (miR-196) rs11614913 and miRNA-146a (miR-146a) rs2910164 are related to susceptibility to ulcerative colitis (UC). Because the previously reported results have been mixed and uncertain, the aim of this study was to perform a meta-analysis and review to assess the relationship between these 2 SNPs and UC risk.

**Methods:**

In this analysis, 5 studies involving 1023 cases and 1769 controls for miR-196 rs11614913 and 4 studies involving 827 cases and 1451 controls for miR-146 rs2910164 were included. Odds ratios (ORs) and 95% confidence intervals (95% CIs) were used to pool the effect size.

**Results:**

A decreased risk of UC was identified in homozygote comparison (GG vs CC: OR = 0.69, 95% CI: 0.52–0.93, *P* = .02), recessive comparison (GG vs CG + CC: OR = 0.74, 95% CI: 0.59–0.92, *P* = .007), and dominant comparison (GG + CG vs CC: OR = 0.79, 95% CI: 0.65–0.97, *P* = .02) of miR-146 rs2910164 in Asian but not Caucasian population. No evidence of an association was shown between the rs11614913 polymorphism and UC risk in allelic, heterozygote, homozygote, recessive, and dominant models in both Caucasian and Asian populations (*P* > .05).

**Conclusions:**

MiR-146 rs2910164, but not miR-196 rs11614913, was associated with a decreased risk of UC in Asian population. However, the results should be treated with caution because of the limited sample size and heterogeneity. Well-designed studies with large sample sizes and more ethnic groups are needed to validate the risks identified in the current meta-analysis and review.

## 1 Introduction

Ulcerative colitis (UC) is a common disease that affects the colon and rectum, and it especially involves the mucosa. The inflammation affects continuous segments of the mucosa but not areas of the intervening normal mucosa.^[1]^ The main clinical manifestations include abdominal pain and diarrhea, which can reduce the quality of life of the patients. It has been suggested that environmental factors, genetic predisposition, and immune dysregulation have important roles in the pathogenesis of UC.^[2]^

UC is a multifactorial, polygenic disease thought to have genetic heterogeneity. According to this hypothesis, different genetic backgrounds may present with different clinical types of the disease.^[3,4]^ Some studies have shown a genetic predisposition through the inheritance of some contributory genetic polymorphisms leading to the pathogenesis of UC.^[5–9]^

MicroRNAs (miRNAs) are small noncoding RNA gene products that adjust gene expression by base pairing with target miRNAs at the 3′-untranslated region, leading to miRNA cleavage or translational repression.^[10–12]^ It has been said that miRNAs are involved in various biological processes including cell proliferation, cell death, stress resistance, and metabolism.^[13]^ Moreover, a recent report has shown that miRNA plays an important role in regulating the pathogenesis of UC.^[14]^ MiRNA has been reported to regulate the nuclear transcription factor kappa b (NF-κB) pathway, thus causing UC. In addition, Wu et al^[15]^ have found that miRs-28-5p, -151-5p, -103-2, -199a-5p, -340, -362-3p, and -532-3p are increased and miR-505 is decreased in the peripheral blood of patients with UC. Moreover, Feng et al^[16]^ have found that miR-126 is increased in UC tissues. MiR-126 may activate the NF-κB signaling pathway through IκBα and promote the development of UC.

Single-nucleotide polymorphisms (SNPs), mutations in the miRNA sequence, may affect miRNA expression and/or maturation.^[17]^ Recently, Wan et al^[18]^ screened for common SNPs in miRNA sequences and verified that 2 SNPs (rs2910164 and rs11614913) located at the pre-miRNA areas of miR-146a and miR-196 are associated with UC. They discovered that miR-146a rs2910164 does not have any correlation with colorectal cancer (CRC) risk. However, it was found that miR-196a2 rs11614913 is associated with the presence of CRC in all genetic models.

There is an increased risk of CRC in patients with UC.^[19]^ Recently, miR-146a rs2910164 has been found to be associated with UC patients, and other miR-196 rs11614913 polymorphisms have been reported to be involved in UC risk as well.^[20–24]^

Although some studies have been performed to explore the association between these 2 SNPs and the risk of UC in various populations,^[20–24]^ the results have been mixed and uncertain. To date, there has been no meta-analysis and review exploring the association between them. Therefore, the aim of this study was to perform a meta-analysis and review to assess the relationship between these 2 SNPs and UC risk.

## 2 Materials and Methods

### 2.1 Identification of eligible studies

An ethical approval is not necessary for a meta-analysis. Two independent investigators did a systematic search in the PubMed and Embase databases. There was no limit in terms of language, and the last search was conducted on March 1, 2018. The following terms were searched: “microRNA” OR “miRNA” OR “microRNAs,” AND “ulcerative colitis” AND “polymorphism.” The reference lists of retrieved articles and recent reviews were searched by hand for further relevant studies.

### 2.2 Inclusion and exclusion criteria

To be included in this meta-analysis, studies had to satisfy the following inclusion criteria: assess the association between miR-146a/196 polymorphisms and UC; be a case–control study; focus on humans; and have detailed genotype data in order to calculate the odds ratios (ORs) and 95% confidence intervals (CIs). Exclusion criteria were as follows: repeat of previous publications; comment or editorial; family-based genealogical studies; and no detailed genotype data. When there were multiple studies from the same population, only the largest study was included. Two investigators independently selected the studies based on the inclusion and exclusion criteria by checking the title, abstract, and full-text. Discussion was used to resolve any disputes.

### 2.3 Data extraction

The following information was collected: first author's name, year of publication, country of origin, genotyping methods, ethnicity, UC criteria, Hardy–Weinberg equilibrium (HWE), numbers of cases and controls, and genotype frequencies in cases and controls for miR-146a and miR-196 genotypes, respectively. The Caucasian or Asian ethnicity was assigned according to the country where the research site was located, namely Caucasians for Europe and Asians for Asia. Two investigators independently extracted the data of the eligible studies. If a dissent existed, they reexamined the original data of the included studies, and discussed and reached a consensus. If a dissent still existed, a third investigator made the final decision.

### 2.4 Methodological quality assessment

The quality of the studies were evaluated according to the Newcastle-Ottawa scale (Table 1).

**Table 1:**
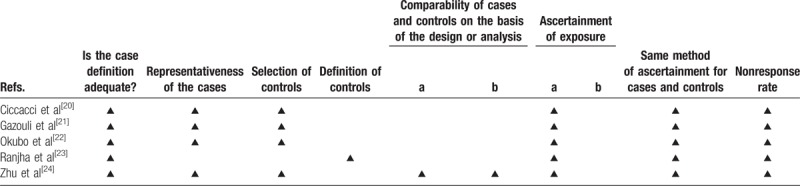
Study quality as determined by the Newcastle-Ottawa scale.

### 2.5 Statistical analysis

A meta-analysis was conducted based on the PRISMA checklist, according to the guidelines.^[25]^ Each study was evaluated by HWE; and after a Chi-squared test with the control group, *P* < .05 was considered to be significantly incompatible with HWE. ORs and 95%CIs were used to assess the association strength between miR-146a/196 SNPs and the presence of UC. Pooled ORs were performed for allelic comparison (miR-146a: G vs C, miR-196: T vs C), the heterozygote model (miR-146a: CG vs CC, miR-196: CT vs CC), homozygote model (miR-146a: GG vs CC miR-196: TT vs CC), dominant model (miR-146a: GG + GC vs CC, miR-196: TT + CT vs CC), and recessive model (miR-146a: GG vs CG + CC miR-196: TT vs CC + CT). A statistically significant level was decided by the Z-test, with a *P*-value < .05 considered to be significant. Heterogeneity was assessed using the *Q* statistic (*P* < .1) and the *I*^2^ statistic (greater than 50% as evidence of significant inconsistencies).^[26]^ Depending on heterogeneity, a fixed effects or random effects model was used to pool the effect size.^[27]^ Sensitivity analyses were performed to assess the impact of each study on each of the findings by omitting each study in turn. Subgroup analyses were performed based on ethnicity. Begg's funnel plots^[28]^ and Egger's regression tests^[29]^ were used to determine potential publication bias. Asymmetric graphs and Egger's tests with a *P*-value < .05 were considered to be a significant publication bias. All statistical analyses were conducted with Stata 12.0 software (StataCorp, College Station, TX). A 2-tailed *P* < .05 was identified as significantly different from specified conditions, where a certain *P*-value was declared.

## 3 Results

### 3.1 Study characteristics

A total of 37 studies were acquired from PubMed and Embase (PubMed: 11, Embase: 26). The literature selection process is shown in flow diagram. Twenty-eight articles were excluded, of which 7 were duplicates, and 21 had no relation to this topic. The remaining nine studies were full-text reviews, and 4 studies were excluded, among which, one was a review.^[30]^ One was not a detailed genotype data study,^[31]^ and 2 others concerned interleukin gene variants of UC.^[32,33]^ Finally, in the present study, 5 eligible case–control studies^[20–24]^ satisfying the inclusion criteria were included. The characteristics of each study are shown in Table 2. Five studies involving 1023 cases and 1769 controls for miR-196 rs11614913 and 4 studies involving 827 cases and 1451 controls for miR-146 rs2910164 were included in the meta-analysis. For miR-146a rs2910164, the subjects in 2 included studies^[20,21]^ were Caucasian, while those in the other 2 studies were Asian.^[22,24]^ With regard to miR-196 rs11614913, 2 studies were carried out on Caucasians^[20,21]^ and 3 studies involved Asians.^[22–24]^ The genotyping methods used included polymerase chain reaction–restriction fragment length polymorphism (PCR-RFLP) and PCR sequencing. The genotype distribution was consistent with HWE in all studies except for 1 study^[18]^ on miR-196 rs11614913.

**Table 2:**
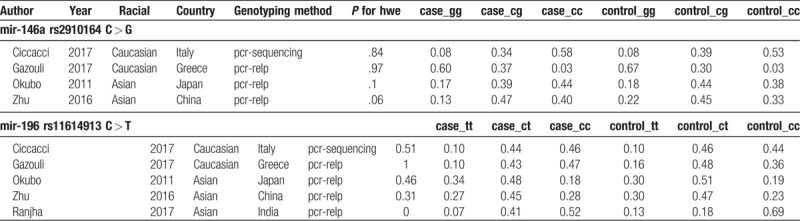
The characteristics of each study were revealed.

### 3.2 Association between the miR-146a rs2910164 polymorphism and the presence of UC

**Figure 1:**
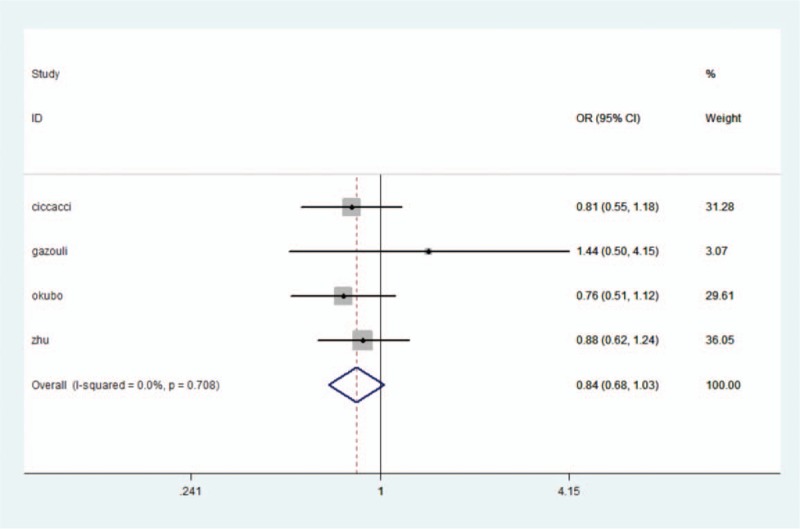
miRNA-146ars2910164 polymorphisms and the risk of UC.

We analyzed the correlation between the miR-146a rs2910164 polymorphism and the presence of UC. In all genetic models, there was no significant heterogeneity by the *Q* test and the *I*^2^ statistic. Therefore, a fixed-effects model was used. No significant association was identified in the allelic and heterozygote model (allelic model: OR = 0.81, 95% CI: 0.71–0.92, *P* = .59; heterozygote model: CG vs CC: OR = 0.84, 95% CI: 0.68–1.03, *P* = .10) (Fig. 1). A decreased risk of UC was identified in homozygote comparison (GG vs CC: OR = 0.69, 95% CI: 0.52–0.93, *P* = .02), recessive comparison (GG vs CG + CC: OR = 0.74, 95% CI: 0.59–0.92, *P* = .007), and dominant comparison (GG + CG vs CC: OR = 0.79, 95% CI: 0.65–0.97, *P* = .02). A trend of reduced risk could be seen (Table 3).

**Table 3:**
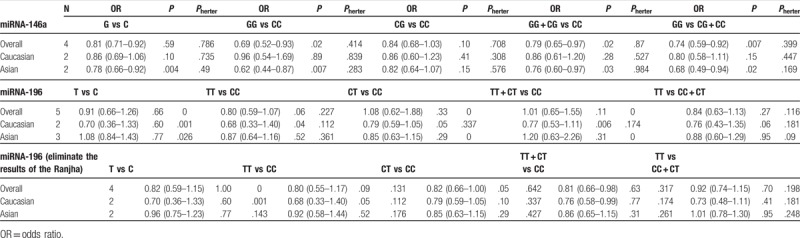
Summary of polled ORs in the meta-analysis.

UC has the highest incidence in Northern Europe and North America, but the lowest incidence in Asia.^[34]^ Because of its association with ethnicity, a subgroup analysis was performed according to ethnicity. In Caucasians,^[20,21]^ significant statistical heterogeneity was not observed. A fixed-effects model was employed for all genetic models. There was no significant association found in any genetic models (G vs C: OR = 0.86, 95% CI: 0.69–1.06, *P* = .10; CG vs CC: OR = 0.86, 95% CI: 0.60–1.23, *P* = .41; GG vs CC: OR = 0.96, 95% CI: 0.54–1.69, *P* = .89; GG + CG vs CC: OR = 0.86, 95% CI: 0.61–1.20, *P* = .28; GG vs CG + CC: OR = 0.80, 95% CI: 0.58–1.11, *P* = .15). A trend of a reduced risk was observed. In Asians,^[22,24]^ homogeneity existed in all 5 genetic models. No significant association was discovered in the heterozygote model (CG vs CC: OR = 0.82, 95% CI: 0.64–1.07, *P* = .15). In addition, a significant decreased risk of UC was discovered (G vs C: OR = 0.78, 95% CI: 0.66–0.92, *P* = .004; GG vs CC: OR = 0.62, 95% CI: 0.44–0.87, *P* = .007; GG vs CG + CC: OR = 0.68, 95% CI: 0.49–0.94, *P* = .02; GG + CG vs CC: OR = 0.79, 95% CI: 0.60–0.97, *P* = .03) (Table 3).

### 3.3 Association between the miR-196 rs11614913 polymorphism and the presence of UC

The association between the miR-196 rs11614913 polymorphism and the risk of UC was analyzed in 5 studies. Because of the heterogeneity, this result should be treated with caution. All models used random-effects models. No association was observed in any comparison.

In order to understand the relationship between ethnicity and disease risk, subgroup analyses were also performed according to ethnicity. Two of the 5 studies were carried out on Caucasians. Significant associations were found in heterozygote model (OR = 0.68, 95% CI: 0.33–1.40, *P* = .04) and dominant model (OR = 0.77, 95% CI: 0.53–1.11, *P* = .006) in Caucasians. In contrast to the results from Caucasian, no significant association was observed in Asians (*P* > .05) (Table 3).

**Figure 2:**
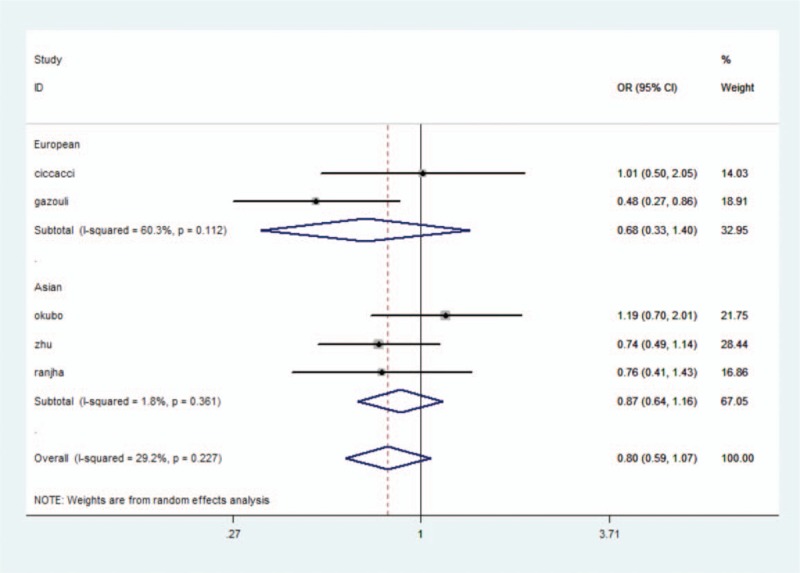
miRNA-196 rs11614913 polymorphism and UC risk.

The genotype distribution was in agreement with HWE in all studies except for one on miR-196 rs11614913.^[23]^ The control group did not conform to HWE, indicating that the sample was not derived from the same group. The sample may not represent a natural population. Therefore, we eliminated this study to observe the effects on the results. Heterogeneity was significantly reduced by excluding this article.^[23]^ A random-effects model was used in the allelic model and homozygote model due to the presence of heterogeneity, and a fixed-effects model was used in the other 2 models. However, no significant association was found in any comparison (*P* > .05) (Table 3) (Fig. 2).

Two of the 4 studies involved Caucasians. Subgroup analysis was performed according to ethnicity. In Caucasians, No statistical association was found in any genetic models in both Caucasian and Asian populations (*P* > .05) (Table 3).

**Figure 3:**
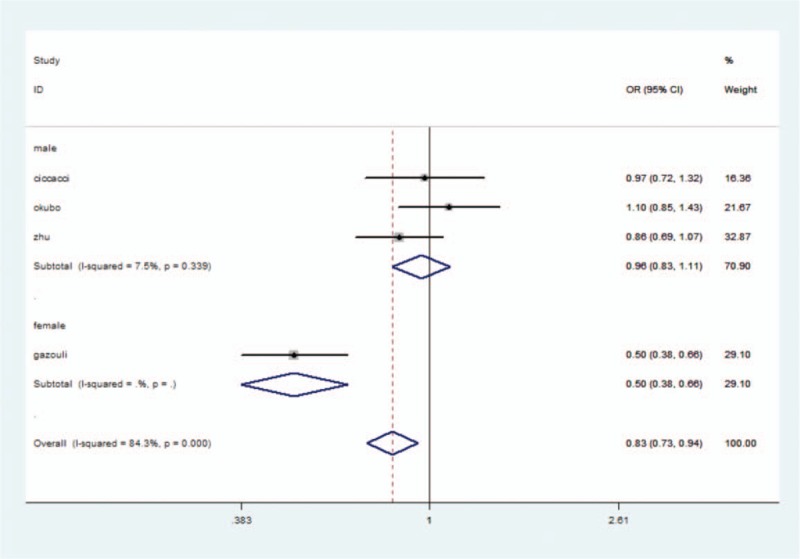
Heterogeneity when sex ratio Gazouli alone as a group, the 3 other studies as another group were separated.

In the study by Gazouli et al, the male/female ratio was 101/109. The proportion of male patients out of the total number was less than 50%. In the 3 other studies, the ratio was greater than 50%. In order to study whether the sex ratio caused heterogeneity, we analyzed the Gazouli et al study alone as a group, and the other 3 studies as another group. This resulted in a reduction of heterogeneity to an acceptable level (Fig. 3).

### 3.4 Sensitivity analysis

Sensitivity analysis was performed by removing a single study from the pooled analysis to explore the effects of individual studies on the pooled results. For miR-146a rs2910164, the results revealed that no individual study impacted the pooled OR significantly, because no substantial changes were found. As for miR-196 rs11614913, sensitivity analysis was also performed by excluding 1 study^[18]^ that deviated from HWE. The results showed that no individual study impacted the pooled OR significantly.

### 3.5 Publication bias

**Figure 4:**
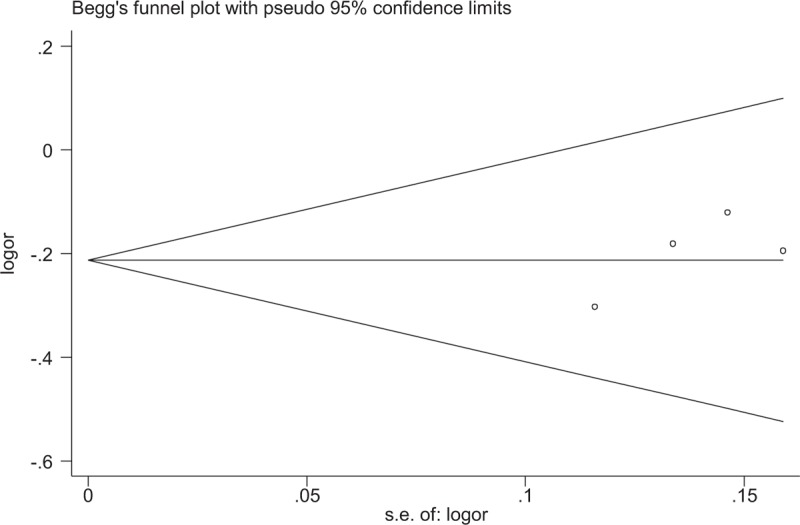
Begg's funnel plot for publication bias analysis for miRNA-146a polymorphism rs2910164 (G vs C).

**Figure 5:**
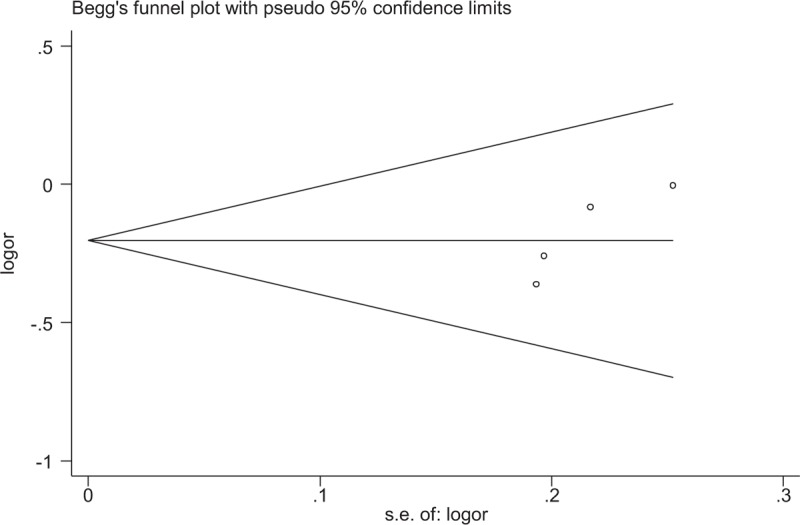
Begg's funnel plot for publication bias analysis for miRNA-196 polymorphism rs11614913 (CT vs CC).

No significant publication bias for the association between miR-146a rs2910164 and the presence of UC was observed by the Begg's funnel plot or Egger's regression test in any genetic model (Fig. 4). As for miR-196 rs11614913, there was no significant evidence of publication bias by excluding 1 study^[23]^ that deviated from HWE (Fig. 5).

## 4 Discussion

We found that the miR-146a rs2910164 polymorphism was associated with a decreased risk of UC in allele comparison, homozygote comparison, recessive comparison, and dominant comparison. Similar results were observed in Asians; however, no evidence was found for the association between miR-146a rs2910164 and UC susceptibility in any genetic models in Caucasians. This finding shows that, in Asians, miR-146a rs2910164 could reduce the risk of UC in some genetic models, but it is not suitable for Caucasians. In regard to the association between miR-196 rs11614913 and UC susceptibility, a decreased risk of UC was observed in the dominant model. This association was significant in Caucasians; but in the Asian population, significant results were not observed in any of the genetic models. These findings indicate that miR-196 rs11614913 could have a protective effect in Caucasians.

MiRNA is a short, single-stranded RNA molecule that regulates gene expression after transcription by binding to complementary sequences in the 3′-untranslated region of target mRNA.^[35]^ MiRNAs have critical roles in regulating diverse processes of cellular growth, such as proliferation, invasion, death, stress responses, and tumorigenesis.^[36]^

Ranjha and Paul^[37]^ have reported that miRNA abnormalities exist in some diseases including UC. In various populations, miRNA polymorphisms can exist as heterozygous or homozygous ligands, in the form of deletion, insertions, translocation amplification, or amplification of chromosomes, resulting in a loss or an increase in miRNA site/function.^[23]^ Ciccacci et al^[20]^ have reported that miRNA SNPs show a significant association with left-sided UC; the rs11614913 SNP was demonstrated to have a protective effect for UC. In addition, Gazouli et al have reported that the rs2910164 CC genotype and C allele are not significantly associated with UC. For the rs11614913 polymorphism, the TT genotype and T allele seemed to have a lower risk for UC.^[22]^ The current results revealed that miR-146a rs2910164 was associated with a reduced risk for UC, except in the homozygote model. We did not find an association between the CC genotype or C allele and UC. There was an association between rs11614913 and a reduced risk for UC in the dominant model. We analyzed the reason for this finding and found that the number of studies included was insufficient and that there was heterogeneity. Five eligible case–control studies for miR-196 rs11614913 and 4 studies for miR-146 rs2910164 were included. If the number of studies is increased, the results could change. If high-quality research is added, the results could be more convincing.

Significant heterogeneity was observed for the association of miR-196 rs11614913 and UC in 3 genetic models. Subgroup analysis was performed according to ethnicity. Heterogeneity still existed in studies involving Asians. In a recent article involving Caucasians,^[20]^ the authors mentioned that the results of the study were different from others. These discrepancies may be due to different SNP frequencies and probably to a different subphenotype distribution in the studied populations. It is suggested that the subphenotype distribution, to some extent, may have contributed to the source of heterogeneity.

The results of our subgroup and sensitivity analyses were stable. A recent research has reported that polymorphisms within the miR-146a sequence caused the additional generation of mature microRNAs from the passenger strands of the miRNA precursor, which can be functionally important because of different target selections.^[38]^ Previous studies demonstrated that the SNP rs2910164 at the premiR-146a (G/C) had a strong relation with many diseases.^[39,40]^ In present combined analysis, we found that a statistical association in 4 genetic models. A decreased risk of UC was shown in allele comparison, homozygote comparison, recessive comparison, and dominant comparison. In the Asian population subgroup analysis, we found a similar result. However, the study on Caucasian populations showed no significant association in any of the genetic models. This finding might have been due to ethnicity, etc. The rs11614913 SNP is located in the pre-mir region, and it was previously associated with several types of cancers.^[41,42]^ A previous study had demonstrated that the expression of mature mir-196a2 may be influenced by the rs11614913 genotype: the presence of the CC genotype decreased the expression of mature mir.^[43]^ The observation that this SNP was also reported to be associated with subclinical phenotypes of UC in the Greek population.^[20]^ However, no statistical association in any genetic model was found in both Caucasian and Asian populations, which indicated this polymorphism might not the susceptible factor for UC risk.

As for sensitivity analysis of miR-146a rs2910164, although the research varied in many aspects, the association could not be driven by a single study. However, for miR-196 rs11614913, sensitivity analysis showed that the study with deviation from HWE conducted by Ranjha et al could alter the association between miR-196 rs11614913 and UC.

The current meta-analysis has several advantages. First, this is the first meta-analysis focusing on the association between miRNA SNPs and risk for UC. Additionally, another miRNA polymorphism of miR-196, rs11614913, was also explored. In addition, according to methodological quality assessments, all of the studies included were of high quality. Moreover, no obvious publication bias was found by either the Begg's funnel plot or Egger's regression test. Finally, there was no limit to the literature search, so selection bias was well controlled.

Although there were considerable efforts to explore the possible association between the 2 SNPs and UC risk, some limitations should be considered. First, the number of studies involving miR-146a rs2910164 and miR-196 rs11614913 was limited, and further analysis was limited by insufficient primary research. Second, 1 study did not conform to HWE. Third, heterogeneity was detected in some genetic models of rs11614913 by excluding 1 study,^[18]^ which deviated from HWE. It is suggested that subphenotype distribution, to some extent, contributed to the source of heterogeneity. This result should be treated with caution. Finally, we did not study different lifestyles to explore the relationship between lifestyle and risk of UC. Therefore, further studies on comparisons between different human populations are valuable and necessary.

In conclusion, our results showed that the miR-146a rs2910164 polymorphism has a decreased risk for UC, except in the homozygote model, especially in Asians, but the conclusion did not extend to Caucasians. While miR-196 rs11614913 polymorphisms may have a decreased association with the risk for UC in the dominant model, the same result was identified in Caucasians, but not in Asians. There was not enough data to adequately confirm the association between UC and miR-196 rs11614913, and the results should be interpreted with caution. Well-designed studies with large sample sizes and different ethnic groups are needed to validate the risks identified in the current meta-analysis.

## Author contribution

**Data curation:** Yi Zhu, Zhongyi Li, Yao Wang.
